# Report of Clinical Observations on Continued (Typhus and Typhoid) Fever

**Published:** 1851-01

**Authors:** Austin Flint

**Affiliations:** Prof. of the Principles and Practice of Medicine, and Clinical Medicine, in the University of Buffalo


					﻿BUFFALO MEDICAL JOURNAL
AND
MONTHLY REVIEW.
VOL. 6.	JANUARY, 1851.	NO. 8.
ORIGINAL COMMUNICATIONS.
ART. I.—Report of Clinical Observations on Continued [Typhus and
Typhoid} Fever. Based on alt' Analysis of Fifty-Two Cases. By
Austin Flint, M. D., Prof, of the Principles and Practice of Medicine,
and Clinical Medicine, in the University of Buffalo.
[Continued from page 401.)
SECTION TENTH.
Symptoms referable to the Genito-urinary System.
The facts, contained in the histories, relating to the subject of this sec-
tion, are quite insignificant. Jn the great majority of the cases, the quan-
tity and other characters appertaining to the urine were unobserved.
This was in consequence of the difficulty of preserving, in a separate
vessel, the evacuation of each patient, and retaining it for my inspection:
a difficulty by no means insurmountable, but which, to my regret, was not
persisting!}’ overcome, the attempt having been commenced, but aban-
doned. The present collection of cases, furnishes no data for determining
what deviations from the conditions of health ordinarily obtain in contin-
ued fever. In a few instances, some unusual symptoms referable to
the urinary system presented themselves, attracting notice, and were
consequently noted. They are as follows :—Copious lithatic deposit was
observed in two Typhoid cases, occuring in private practice. In these cases
this deposit took place during the whole course of the disease.
In the history of one case of Typhus, it is noted occasionally during the
progress of the disease that the urine was high colored, and without sedi-
ment, but two days before convalescence was pronounced, the secretion
became more abundant, and deposited a copious lateritious sediment.
In one case of Typhoid inprivate practice, there was, fora time, inability
to void urine, requiring the catheter. The inability in this case did not
proceed from unconsciousness, the patient experienced fully the desire to
urinate, but was unable to accomplish the act. This was early in the dis-
ease. and may, perhaps, have been owing to anodyne remedies, which, as
is well known, occasionally are attended with such an effect.
In another Typhoid case in private practice, there was some difficulty in
evacuating the bladder, for a day or two, which, perhaps, was due to the
same cause.
In several cases, it was noted that the urine was passed in bed. This
was owing to the same mental indifference which accounts for the evacu-
oation the bowels in bed; and the two events generally co-existed.
These few and meagre observations are all that I have to report.
SECTION ELEVENTH.
Duration of the disease. Circumstances attending Convalescence. Seque-
la!. Mode of dying. Fatality.
Duration.—In order to determine, accurately, the duration of the
febrile career, it is necessary that the periods of its commencement and
termination should be fixed'with precision. It has been seen, in a previous
section, that it is not easy to decide, in all cases, upon the exact time when
the access, or forming stage closes, and the fever begins. The circum-
stance selected to denote the period of commencement, in the present
analysis, is, confessedly, wanting that degree of precision which could be
desired. It is, in a measure, arbitrary, and is adopted only because, in the
opinion of the writer, it supplies, in the majority of cases, a criterion as
accurate and as readily available, as any that may be fixed upon. The
circumstance referred to, is a sense of disease or debility sufficient to
lead the patient to desire to take to the bed. This marks the date of the
fever proper, or of the second stage if the access be included among the
stages of the disease.	%
blow, what shall serve as the index to the day of convalescence? The
rule adopted by Louis, is to consider the day of convalescence that on
which the patient begins to take some solid food. In following this rule,
however, it is plain that he determines the fact of convalescence, prior to
the patient’s taking food, from other circumstances. That is to say, when
the patient is distinctly convalescent he allows solid food for the first time,
and the latter is, therefore, merely an indication of his decision, not, neces-
sarily, a criterion of the fact of convalescence. The applicability of this
rule will involve the withholding of solid food prior to convalescence, on the
one hand, and that it should be invariably given so soon as convalescence
is pronounced, on the other hand, both of which provisions may not be in
accordance with the course pursued by all practitioners. Notwithstanding
these objections, I am not aware that a better criterion has been suggested.
But instead of following the rule laid down by Louis, it appears to me
preferable to do what it would seem Louis virtually does, viz: decide on
the day of convalescence from an ensemble of circumstances. If the
febrile movement, as determined by the heat of skin, acceleration of pulse,
etc., have ceased, clearness of the intellect returning, with refreshing sleep,
and the patient has a desire for, and relish of food, he may be pronounced
convalescent. Some one or more of the above conditions, in some instan-
ces, may be wanting, and, still, the other circumstances be such that con-
valescence may properly be declared. Judgment, and some experience are
requisite to decide correctly; and, with every qualification on the part of
the observer, it will not infrequently be a matter of some doubt as to the
particular day which should limit the termination of the febrile career. Dif-
ferent practitioners would not fix upon the same day in all cases, owing to
differences in the mode of estimating the circumstances upon which the
opinion is based. Perfect exactitude and entire uniformity, in short, as re-
spects this point, are not practicable: and yet, sensible physicians, in the
majority of instances, will act with sufficient correctness for all practical
purposes. The exercise of judgment in a similar way, would answer
equally well in designating the commencement of the disease, without re-
course to an arbitrary event like that which the writer has adopted. But
there is this mateiial difference in the two instances, viz., it very frequently
happens that cases do not come under observation at their commencement,
and hence, it is desirable to fix upon something which will serve to mark
the date at a period when we have nothing to rely upon but the history
given by the patient or friends. Under these circumstances we can almost
always ascertain at what time the patient took to the bed, when we are
able to obtain very little information of the symptoms, and history in other
respects. On the other hand, a patient does not pass from our observation
usually, until he is considered convalescent; hence, there is not the same
necessity, or advantage, in determining the time of convalescence by refer-
ence to an arbitrary standard.
It was an ancient belief, which is not yet entirely obsolete, that there is
an intrinsic tendency in Continued Fever to terminate after the lapse of
a certain numbers of days, dating from the commencement of the disease.
That the doctrine of critical days, as these days were termed, originated in a
fancied virtue inherent in the days, or numbers by which they were desig-
nated, is evident from the foregoing considerations. The difficulty of de-
ciding the duration, to a day, in a large proportion of cases, renders it im-
practicable either to establish, or disprove, by observation, that doctrine.
This, however, has been attempted within a few years. Dr. Welch, of
Edinburgh, Scotland, enumerated the days in 690 cases, collected from dif-
ferent sources, and he found that, of this number, 470 ended on some of
the days considered critical. Subsequently Dr. Davidson, of the same
place, analyzed a collection of cases, considerably less in number, with re-
ference to this point, and arrived at different results. Statistics by differ-
ent persons, with an equal allowance of good faith, may be expected thus
to differ. The termination, as well as invasion of the disease being seldom
so well defined but that one of two or three days might be selected, a dis-
position to credit or discredit the doctrine of critical days would afford a
bias which would hardly fail to determine the preference of critical or non-
critical days according to the pre conceptions of the observer.
Insdetermining the duration of the disease, then, in the cases under in-
vestigation, rigorous accuracy is not claimed, but only an approximation
thereto, sufficient for all practical purposes exclusive of the doctrine of cri-
tical days.
In about one half of the hospital cases (17 of 38) the histories do not
contain a definite statement of the commencement of the disease, i. e., of
the time the patients had been confined to the bed prior to entering the
hospital. In many instances it was impracticable to obtain information on
this point, and proper effort was not always made to obtain it. Patients
were received at different periods of the disease, and hence, the space of
time from the date of their admission to the date of convalescence, is not a
fair representation of the duration of the disease. In a considerable num-
ber of cases, however, it may be supposed that the differences in the time
that had elapsed before entering the hospital would compensate for each
other, and, the cases of different groups might be considered uniform in
this respect Hence, the cases of the two types may be compared as re-
gards the duration from the date of admission. The time of convales-
cence was generally fixed, being determined by so marked an abatement
of the symptoms as to denote that the febrile career was at an end. In a
few cases, however, this was impracticable, owing to the existence of com-
plications, continuing after the febrile career was ended, symptoms of both
being so intermingled, that chronological limits of the latter were not dis-
cernible.
Another point of inquiry relates to the time of remaining in hospital, i. e.,
the number of days, or weeks, from the date of admission, to the date of
discharge. Here various causes are operative aside from disease, and the
amount of debility remaining after convalescence is established. Some pa-
tients are anxious to leave the hospital as soon as possible, and do not re-
main as long as prudence would dictate; others, having no better asylum,
are desirous of staying as long as possible. Still, on the principle of com-
pensation for differences, the cases of the two types may be considered uni-
form in this particular, and a comparison instituted.
The facts contained in the histories of the cases under examination rela-
ting to the foregoing objects of enumeration and comparison, are presented
in the following tables :—
TABLE EXHIBITING DURATION, ETC., IN EIGHTEEN HOSPITAL TY-
PHOID CASES.
1 2 3 4 W1 ~8 976711213 1415161718
No. of days from
time of taking to
bed, to convales-
cence.	18	1814	25 31 14 Total 120.
---------------------------------------------Mean 20.
►—
□
o-
a
No. of days from	o
admission to hos-	3
pital, tn date of	s'
convalescence.	8,8 12 13 12 13 14 10	15 32* 14 21 6 9 £ Total 187.
		- Mean 13 5-14.
No. of days from------------------------------------------------3
date of convales-
cence to date of	~
discharge,or date
of last record. 23	11 9 4 9 4 7 5 6	7	30 13	7 ? Total 135.
—--------------------------------------------S Mean 10 5-13.
Fatal cases.	g-
No. of days from
taking to bed.	26	10
Do. from admis-
sion.	4 15	G	10
Length of time	15 19 17 16 22 16 20 19 16 6221	4434 16 16 Total 258.
in hospital.	| J	Mean 19 13-15.
* Complicated with pneumonitis.
TABLE EXHIBITING DURATION IN TWELVE TYPHOID CASES IN
PRIVATE PRACTICE.
————;p'a-3	4	5~g	7~8~~9~ 17TH 12
No. of days from
time of taking to
bed, to date of about about about
convalescence. 16 11 18	21	28 15	11 10 14	9	5	9 Total 149.
\ Fatal. Fatal. Mean 14 9-10.
TABLE EXHIBITING DURATION IN 12 HOSPITAL TYPHUS CASES.
1	2	3	4	5 6 7	8	9	10(11 12
No. of days from Fatal.	Fatal
data of taking to 13th	on the
bed to date of day af-	7th
convalescence.	ter in-	day.
vasion. 19	19	9	13	14 Total 74 days.
__________________________________________________Mean 14 4-5.
No. of days from	Fatal
admission to hos	8th	Fatal
pital to date of day.	on the
convalescence. 8	12	14	18 9 13 3ddav. 13	14 17 10 I otal 131.
__________________________________________________Mean 11 10-11.
Com-	Re-
plica-	main’d
ted	in hos-
with	pital 6
paroti-	weeks,
tie.	Trans-
ferred
to sur-
No. of days from	gical
date of convales-	depart-
cence to dismis-	ment
sal from hospital	for es-
or date of last re-	charon
cord.	16	37	5	9	sacrum 16 21 11 Total 115.
--------------------------------------------------Mean 16 3-7.
Length of time 8	28	51	23	22	30 38 21 Total 221.
in hotpital.	J	_____ Mean 27 5-8.
TABLE EXHIBITING DURATION IN EIGHT HOSPITAL CASES OF
DOUBTFUL TYPE.
1 2 3	4	5	6	7	8
No. of days from
time of taking to
bed to date of
convalescence. 9 12 14	14	9	5 Total 63.
___________________________________________________________________Mean 10J.
No. of days from	Fatal
date of admis-	23rd day,
sion to hospital	complica-
te date of conva-	ted with
lescence.	6 10 9 parotitis. 5	9	9	5 Total 53
	Mean 7 4-7
No. of days from	Remain-
date of convales-	Parotitis Parotitis ed in hos-
cence to date of	superve- Buperve-pital. Sis- Sister
discharge from	ned. ned.	ter of of
hospital, or of	Charity, charity
last record. 5 5 17	35	27
__________________________________________________Mean 17 4-5.
Length of time 11 15 26	40	36	lotul 128.
jn hospital._______________________________________________________Mean 25 3-5.
The shortest term of duration, as will be perceived by consulting the ta-
bles, was five days. In two cases, one being in private practice, and one
among the hospital cases of doubtful type, this was the length of the fe-
brile career. The history in each of these cases leaves no doubt as to the
fact that the disease was Continued Fever. In both cases the fever was
of a very mild grade, but the diagnostic features were unequivocal. In
both instances it seemed most rational to attribute the origin of the disease
to contagion. There were exceptional cases as respects duration. In no
other case did the fe^er run its course in a less period than nine days, and
in all but five instances, the duration was longer. The maximum of dura-
tion was twenty eight days, the case occurring in private practice. This
case was treated homoeopathically for ten days before coming under my
observation, the patient being kept on rice water for diet.
It will be observed that the average duration of the Typhoid cases in
private practice is considerably less than the average of those of the same
type in hospital, 14 9-10 being the mean number of days in the former, and
20 the mean number of days in the latter. I can offer no explanation of
this disparity. It should, however, be considered that the cases in hospital
in which the criterion for determining the commencement of the disease was
ascertained, are few in number—being only six. On comparison of the
average duration of the hospital cases of Typhoids with that of the cases of
Typhus, the latter are found to have a shorter career, the mean number
of days being 14 4-5, while the mean number of days of the former, as just
stated, is 20. This disparity accords with the results of analyses by
others.
The disparity is less, if we compare the hospital cases of the two types as
respects the duration from the time of admission into hospital to the time
of convalescence. The mean number of days of the Typhoid cases from
the date of entrance to the date of convalescence is 13 5-14, while the mean
number of days of the Typhus cases is 11 10-11. The probable explana-
tion of this result is, that in cases of Typhus the symptoms sooner assume
a degree of gravity to lead the patient or friends to seek hospital relief.
Although the career of the disease is shorter in the majority of cases,
in Typhus, patients are nearly as long in hospital before convalescence, be-
cause they enter sooner than in cases of Typhoid.
Again, on comparing the hospital cases of the two types as respects the
duration from the time of convalescence to the date of the last record, or
discharge, the disparity is in favor of the Typhoid type, the mean number
of days being in the latter 10 5-13, while in the Typhus type it is 16 3-7.
This result would go to show that the system recovers sooner from the el-
ects of Typhoid than Typhus fever, or, in other words, it shows that the
latter form of fever, although shorter in its career, produces a graver influ-
ence on the organism. The length of time in the hospital in cases of the
two types, corresponds with the above result, the mean number of days in
Typhoid being 19 13-15, while in Typhus it is 27 5-8.
It remains to notice the duration in the cases ending fatally. Of the five
fatal cases of Typhoid type, the duration of the disease in each was as fol-
lows:—after the attack, nine days in two cases; twenty-six days in one case;
ten days in one case. In the other cases the dates of the invasion were
not precisely ascertained. The duration after admission into hospital of the
latter cases, were as follows:—three days in one case; six days in one case.
Mean duration of time the cases were under observation, 8 1-5. Of the
three fatal cases of Typhus the duration in each was as follows:—three
days in one case; thirteen days in one case; seven days in one case. Mean
duration of time these cases were under observation, 6 1-3. The mean pe-
riod of duration when the disease proved fatal, is considerably greater in
the cases of Typhoid than in those of Typhus, being 13^ in the cases of
the former in which the date of the attack was ascertained, and 8 in the
cases of the latter type. In so far as these few cases afford data for re-
sults, then, Typhus, when it proves fatal, runs a shorter career than Ty-
phoid, a conclusion which accords with the observations of others.
Of the two fatal cases of doubtful type, the duration was in one, eight
days, and in the other, twenty-three days,
There are several points of inquiry connected with the subject of dura-
tion, in addition to those which have been noticed. One of these is the in-
fluence of age upon duration; another is the influence of early or late re-
ception into hospital; the effect of seasons is another, etc. The number of
cases in this collection is not sufficiently large for enumerations with refe-
rence to these questions.
Circumstances attending convalescence. The circumstances attending
convalescence are of interest with reference to the pathological inquiry in
how far they may be considered to have contributed to the favorable ter-
mination of the disease; in other words whether, or not, they are critical
in their character. Of the ten Typhoid cases in private practice which
terminated favorably, convalescence in one case was preceded by profuse
perspiration for several nights; and in one case it is stated that convales-
cence was accompanied by profuse perspiration. In one case diarrhoea,
which had existed moderately during the progress of the disease, was some-
what increased at the time of convalescence. Of the remaining seven
cases, it is stated in four that no evacuations occurred which could be re-
garded as critical; and in three there is nothing stated on the subject. The
occurrence of an event which was suspected to be critical, would hardly es-
cape being recorded; and, hence, it is fair to infer that in all the cases ex-
cept the three first mentioned nothing of the kind was observed.
In five of the above ten cases, convalescence was established by a grad-
ual abatement of symptoms, and the histories of the remaining five cases
do not furnish data for determining whether the disease ceased suddenly,
or not. In the latter group are included the two cases in which perspira-
tion occurred at, or near the time of convalescence.
Of the fourteen Typhoid hospital cases which terminated favorably, con-
valescence was accompanied by perspiration in two. In one of these cases
the perspiration was profuse. In one case diarrhoea continued, but not in-
creased. In one case, the contents of a small abscess on the thigh were
discharged on the day preceding that on which convalescence was pro-
nounced. Nothing is stated of any event which might be supposed to be
critical in the histories of any of the remaining cases. In two cases, the
occurrence of convalescence was apparently postponed by the existence of
pneumonitis, and in one case, the same was due to parotitis.
Of the ten Hospital cases of Typhus which terminated favorably, con-
valescence was preceded or accompanied by perspiration, in four. In none
of the remaining cases does the histories contain aught which could be
regarded critical. In two cases, pneumonitis co-existed, and continued after
the febrile career had ended; and in one case, the same was true of pa-
rotitis.
Of the seven cases of doubtful type which ended favorably, convales-
cence was accompanied by profuse perspiration in one case. Pneumonitis
existed at the time of convalescence in one case.
In the preliminary analysis of the Hospital cases, pains -were not taken to
ascertain whether the febrile career ended suddenly, or by a gradual abate-
ment of the symptoms.
The above results show a small number of cases in which there are any
appreciable circumstances connected with convalescence, that may be sup-
posed to have contributed to the favorable termination of the disease.
The instances in which diarrhoea continued, are hardly entitled to consid-
eration in this point of view. Perspiration, which occured in nine of forty-
two cases, it may at first be supposed, was a critical event. But when it
is considered how large is the proportion of cases in which sweating occurs
during the career of the disease, prior to its occurrence in connection with
convalescence, (viz. in twenty-three of forty-seven^ there does not seem to
be sufficient ground to attribute to it much, if any, agency in determining
the probable termination. From the frequency of its occurrence, irrespec-
tive of convalescence, it might, perhaps, be expected to occur at, or near
the time of convalescence, in about one-fifth of the number of cases, by
the law of probabilities, having no connection with the convalescence be-
yond that of coincidence in time. Assuming that there does exist some
connection, other than this, it would by no means follow that the perspira-
tion contributed to the convalescence. The former may be an effect of
the latter, instead of being involved in its causation, or both maybe effects
of the same favorable change in the morbid actions constituting the dis-
ease. To discuss the latter points, would be to engage in speculative in-
quiries ; but, as it seems to me, a comparison of the ratio of the occurrence
of perspiration in Continued Fever, without any amelioration of symptoms,
with the ratio of its occurrence, at or near the time of convalescence, suf-
fices to disprove the supposition that there exists any such connection
between these events.
Sequelce.—Under this heading, I will include occurrences worthy of note,
which transpired from the time convalescence was established, up to the
periods when the cases passed from observation. The system, after the
febrile career, is liable to disorder, or disease, of various kinds, and, at some
times and places, it has been observed that particular affections are apt to
ensue, showing a predisposition to certain sequelae derived from the disease
through which patients have just passed. No special tendency to subse-
quent affections characterized the cases in this collection, and, in the great
majority of instances, convalescence was not retarded by any untoward
event. The occurrences which were noted, are as follows:—
In the group of cases in private practice, convalescence was marked by
unusual characters in but a single case. In the case referred to, the pa-
tient, for several days, exhibited a delirous excitation of ideas, and vari-
ous delusions, the most prominent of which was, that he bad acquired great
wealth, that he owned steam boats, &c., and, among other extravagances,
he entertained the strange conceit that he had remunerated his medical
attendant with a munificent fee 1 This patient, during the febrile career,
did not manifest marked delirium, replying coherently to questions, but he
recollected very little that transpired in the course of his illness. The de-
lirum above mentioned became developed after all the other symptoms
denoted convalescence, and indeed when he was able to sit up a portion of
the day. The delusions suddenly vanished. He said he thought the mat-
ter all over, and discovered the fancifulness of the ideas, which had, for a
few days, afforded him so much satisfaction. The discovery did not induce
any depression, he continued to convalesce rapidly, and has enjoyed excel-
lent health for the two years that have now elapsed since his recovery.
May not the delirium, in this case, have been due to an exuberence of that
which occasions the pleasurable sensations incident to returning health ?
Of the Hospital Typhoid cases, in one the formation of a small quantity
of pus near the rectum occured during convalescence. The parts healed
readily after the purulent discharge. In one, diarrhoea and indigestion
occurred, which were of short duration, and were probably due to impru-
dence in diet.
In one, hysterical paroxisms occurred. In one, the submaxillary gland
became swelled, but the swelling subsided and disappeared in a few days ;
and subsequently formation of pus occured on the thigh just below the
trochanter, and in the axilla. These abscesses discharged for several days,
and the part3 then healed, the patient leaving the hospital quite well, thirty
days after convalescence was pronounced. In one, perspiration occurred
for several nights, and on one night slight delirium.
Of the Hospital cases of Typhus, in one the patient seemed dull, and
apparently experienced some difficulty in arranging his ideas. In one
several furunculi on the legs appeared in succession, and, in the
same case, during the time of convalescence, the gums were spongy,
and bled for a few days. The latter was not the effect of mercurialization.
In one, a furunculus appeared on the ankle, several days after convales-
cence, and the patient complained of pains in the loins, which were relieved
by the application of a sinapism. In one, parotitis followed convalescence,
and this was succeeded by erysipelas of the face.
Of the cases of doubtful type, in one, cough and substernal pain occured,
relieved by a blister. In one, vomiting, probably occasioned by impru-
dence in diet. In one, considerable febrile movement was observed on one
day; and in one, parotitis commenced eight days after convalescence was
pronounced.
The foregoing list shows nothing like uniformity in the few sequelae
which were noted, nor were any of the subsequent affections of serious
import. It is to be borne in mind that the histories, for the most part,
embrace only the time elapsing after the cases came under observation,
up to the date of the discharge. Most of the patients were lost sight of,
after leaving the hospital. In the instances, which are exceptions to this rule,
it is not recollected that any have experienced disease up to the present
time.
In none of the cases did relapse occur. I have never witnessed what
might properly be called a relapse, after the career of Continued Fever was
ended. A recurrence of febrile movement from persistence of some of the
complications of the disease, or supervening affections, has been observed,
but not a recapitulation of the phenomena necessary to constitute Contin-
ued Fever.
I	have not noticed in this connection the instances in which complications
and symptoms, developed during the career of the fever, continued, re-
tarding recovery, and modifying the symptoms of convalescence. This
was the case with respect to pneumonitis in several cases, and parotitis.
Deafness, congestive redness, diarrhoea, coated tongue, tremulousness of the
tongue, acceleration of the pulse, etc., were observed in convalescence, as
well as during the febile career, but this has been already stated under
divisions treating of these affections and symptoms distinctly.
Mode of Dying.—The mode of dying in fatal cases of Continued Fever,
is not uniform. I will consider the diversities in this particular, as arran-
ged into two kinds—first, dying by apnoea, and second, by asthenia.
Death by apnoea may be occasioned by any cause acting directly to sus-
pend respiration. The cause may be in the respiratory organs, but oftener
it is situated at the cerebral centre, destroying the instinctive want of res-
piration necessary to maintain the involuntary movements involved in that
function. Death by asthenia is occasioned by causes acting more directly
on the circulatory forces, affecting the vis nervosa upon which the contrac-
tile property of the heart depends; or depriving the heart of its proper
stimulus, the blood, either by diminution of its quantity, or impairment of
its quality. These two general modes are not infrequently commingled
in fatal cases of Continued Fever. Asthenia may contribute to induce a
condition of the cerebral centre, in consequence of which, death occurs
from apnoea sooner than it would have resulted from the former mode
alone. Both modes are so conjoined in some cases, that it is not easy to
decide which really determines the fataljssue. I will proceed to enumer-
ate the fatal cases, giving the duration of each, and the mode of dying
which appears most probable from the histories. The circumstances con-
nected with the last moments of life are not always detailed, so that the
mode of dying cannot be asserted with positiveness in all instances.
The fatal cases in private practice :—
1	(Typhus,) Death on the 4th day, by asthenia.
2	(Typhoid,') on the 9th day, by asthenia.
3	(Doubtful type,) Sth day, by asthenia.
17 (Typhoid,) 9th day, by apnoea.
The fatal Hospital cases :—
1	(Typhoid,) Death on the 3d day after admission, previous duration
not ascertained, by asthenia.
2	(Typhoid,) on the 15th day after admission, and the twenty-sixth
after the date of the attack, by asthenia.
3	(Typh oid,) on the 6th day after admission, previous duration not
ascertained, by apnoea.
4	(Typhoid,) on the 10th day after admission, and date of attack, by
asthenia.
5	(Typhus,) on the Sth day after admission, and 13th after date of
attack, by apnoea.
6	(Typhus) on the 3d day after admission, and seventh after date of
attack, by apnoea.
7	(Doubtful type?) on the twenty-third day after admission, previous
duration not ascertained, by asthenia.
Fatality.—The ratio of mortality in fifty-two cases, upon which this
report is based, is a fraction over 21 per centum. In the Hospital cases,
it is 18 8-19 per cent., while in the cases in private practice, it is 28 4-7
per cent.* The ratio of mortality is a fraction greater in the cases of
Typhus, than in those of Typhoid. The proportion of instances in which
the disease proved fatal, is not large, judged by the average rate of mortal-
ity of this disease, and it is proper to state that the proportion of fatal
cases would have been less, if all the Hospital cases of Continued Fever
had been included in the collection. A few cases, which were probably
ca ses of Continued Fever, were rejected because there seemed some
room for doubt as to the correctness of diagnosis, and a few were excluded
inconsequence of meagreness of the histories; but every fatal case of the
disease is embraced in the analysis.
* In the remarks preliminary to Section First, (page 136) the ratio of fatality is erro-
neously stated. The reader is requested to make the correction.
SECTION TWELFTH.
Examinations after death.
In the few examinations which were made after death, the object was
not to ascertain the appearances of all the organs, in order to gather from
this source a complete assemblage of negative as well as positive facts.
Time and opportunity were wanting for so minute and thorough an inves-
tigation as would be requisite for that object. The more important of the
vital organs, in some cases, were inspected ; in some instances the dissec-
tion was limited to the display of the lesions which are most characteristic
of this disease. In the four fatal cases in private practice no examinations
after death were made.
Of the seven fatal cases in Hospital, examinations more or less extensive
were made in five. The morbid appearances disclosed in these five cases,
respectively, were as fellows:
No. 1. [Typhoid.} Autopsy twelve hours after death.
Head.—Moderate adhesion of dura mater. Arachnoid membrane dia-
phanous; a little scrum beneath the membrane at dependent portion of
brain. Some effusion at base of brain, quantity could not be estimated,
being commingled with blood which escaped on removing the brain from
the skull. Large veins between convolutions, congested. A considerable
number of red points on section of cerebral substance. Ventricles empty.
Consistence of brain’s substance normal.
Chest.—Slight, old adhesions over small space in right chest. Several
ounces of sanguinolent effusion (say 5 or 6) in left chest. No adhesion in
this chest; no lymph. Pleural membrane presented nothing to attract no-
tice. Lungs on both sides free from morbid appearances, presenting the
amount of hypostatic congestion generally found in autopsical examinations;
crepitating throughout.
Heart.—Slight effusion of transparent serum. Organ rather below nor-
mal size. Left ventricl® empty. Left auricle contained a small quantity
of fluid blood. Right ventricle contained fluid blood, with some small, 6oft
coagula. Right auricle moderately distended with blood, mostly fluid.
Abdomen.—Colon and ccecum greatly distended with gas, small intes-
tines moderately so. Dividing the ileum close to the ccecum, and, length-
wise, for several feet upward, the glands of Peyer were found to be enlarged,
projecting two or three lines above the surface of the surrounding mucous
membrane. From fifteen to twenty patches were counted, the enlarge-
ment diminishing, progressively, upward. No ulcerations, nor discoloration
perceptible. Mucous surface appeared healthy. Numerous enlarged soli-
tary glands visible, some as large as small peas. Mesenteric glands, in
portions of mesentery corresponding to diseased glands of Peyer, greatly
their enlarged. At lower portion of ileum they were as large as filberts;
enlargement was less and less, in correspondence with the diminution of
the size of Peyer’s glands upward, and they ceased to be visible at a point
corresponding to the limit of the latter. On section on the Mesenteric
glands, some presented a red, and some a white color; none were found to
contain pus or other fluid.
Stomach.—Presented punctuated redness, or ecchymoses. No capilli-
form redness. Size normal. Mucous membrane softened. Several ulcer-
ations varying in size and form, the largest half an inch in length, and
three or four lines in width, superficial, apparently having penetrated only
the mucous coat. These appearances were limited to the larger curvature.
The organ at this part v as easily torn, a rent occurring in removing it from
its vascular splenic attachments.
Spleen somewhat enlarged, but not softened.
Liver congested. Otherwise normal.
This case was characterized by active delirium, which persisted through
the whole career of the fever. The absence of the evidence of encephalic
inflammation in connection with this fact, is deserving of note. A report
of the case in full, together with the above autopsical appearances, is given
in the Buffalo Medical Journal, vol. IV, page 487, et seq. (No. for January
1849.)
No. 2. (Typhoid.) The examination in this case was limited to the abdo-
men, and even here was cursory. The omenturn was adherent to the intes-
tines, and the intestinal convolutions were adherent to each other. These
adhesions, for the most part, were easily ruptured, but in some situations
they were so firm that the force required to separate the adherent parts,
occasioned rupture of the tube. In several instances, in separating the
parts united by firm adhesions, small quantities of purulent matter es-
caped.
On longitudinal section of the intestinal tube, recently cicatrized ulcera-
tions of Peyer’s glands were apparent near the ileo-coecal valve. One of
these was as large as a quarter of a dollar. The abrupt termination of the
mucous membrane formed a ridge surrounding these spaces, defining
their boundaries. The parts that had been ulcerated were coveied with a
delicate, smooth surface like serous membrane. The glands of Peyer,
for several feet above the coecum, presented ulcerated patches varying in
size and form, which were mostly cicatrized as just described, but in some
of those situated higher in the tube, the healing process was not com-
pleted. The mesenteric glands were enlarged, but none larger than a
large pea.
Death occured in this case fifteen days after admission, and twenty-six
days after the date of the attack.
A report of this case is contained in the Buffalo Medical Journal, vol. V,
page 271.
The case was interesting in consequence of its affording a fine illustra-
tion of cicatrization of intestinal ulcerations completed, and in progress of
completion. The patient died evidently of chronic peritonitis.
No. 3. (Typhoid.} Autopsy fifteen hours after death.
Head. Considerable congestion of the veins of the superficies of the
brain, and numerous red points on section of the cerebral substance. Some
effusion into arachnoid sac, precise quantity not ascertained. No effusion
into ventricles. No softening of brain. Not the least opacity of arachnoid,
nor effusion beneath it.
Abdomen. Colon distended with gas ; small intestines collapsed. The
latter contained a small quantity of thin yellowish matter. Peyer’s
glands hypertrophied, so as to project above the surrounding mucous sur-
face ; especially an elliptical patch near the ccecum. The ileum in the
space of about three feet above the ccecum presented several elevated
patches of different sizes. No ulcerations. Mesenteric glands correspond-
ing to the lower portion of intestines, much enlarged. No lumbrici. The
stomach, on its internal surface, presented several patches of ecchymoses.
The mucous tunic was not softened, but easily detached from the middle
coat.
Spleen small, and did not seem softened.
Liver presented nothing worthy of note.
Bladder much distended with urine. Other organs not inspected.
The history of this case from the time it came under observation, up to
the termination, is given in full in this report—see page 203. It was re-
garded as a case of cerebral disease prior to the autopsy. The case affords
another illustration of great predominance of cerebral symptoms without en-
cephalic inflammation. It is also worthy of note in this case, that not only was
diarrhoea absent, but costiveness existed in an extreme degree, resisting
several drops of croton oil. It illustrates the fact that the absence of diar-
rhoea, and the existence of obstinate costiveness do not afford grounds for
an inference that the glands of Peyer are unaffected.
No. 4. (Typhoid?) Autopsy, about fourteen hours after death.
Abdomen.—Peyer’s glands hypertrophied, and, nigh to the ccecum, a
large ulceration of the size of half a dollar. Enlargement of the mesen-
teric glands, greatest at the portion of mesentery corresponding to the
lower portion of the ileum, and gradually diminishing in an upward direc-
tion. Hypertrophy of Peyer’s glands gradually diminishing in degree, in
ascending the tube. Spleen softened. Liver presented no morbid ap-
pearance. Other organs not inspected.
This case terminated fatally on the tenth day, by asthenia. The his-
tory of this case affords an illustration of the coexistence of costiveness
with follicular disease and ulceration. The febrile career was not charac-
terised by any peculiar circumstances.
No. 5. (Typhus.)
Head.—Moderate adhesion of dura mater to calvarium. Slight flow of
serum on dividing the arachnoid. Arachnoid membrane transparent.
Veins situated between the convolutions, injected. A little serum at the
base of the brain. A few red points on section of the cerebral substance.
No softening. No effusion into ventricles.
Chest.—The lungs anteriorly presented nothing unusual. Posteriorly,
they were deeply congested, and solidified. This was greater in some
lobules than in others. Some lobules emphysematous.
About an ounce of clear serum in pericardium. Left auricle and ven-
tricle contained a small quantity of fluid blood, without coagula. Right
ventricle contained a small, soft coagulum, a portion of which was white;
no fluid blood. Size and consistence of heart normal.
Abdomen. Ccecum and colon greatly distended with gas, external appear-
ance of intestines normal. On dividing the ileum, the patches of Peyer,
for the space of several feet above the ccecum, were distinctly visible,
numbering, in all, about a dozen. The lowest were somewhat softened.
The appearance of the mucous membrane, otherwise, normal. A little
thin yellowish matter, adherent to the mucous membrane. Spleen not
enlarged, but softened. A few mesenteric glands at the lower portion of
the mesentery, enlarged to the size of a small pea. Liver presented noth-
ing abnormal. Stomach normal in size, mucous lining of a dingy brown
color, notably thickened, and somewhat softened. No redness, nor vas-
cular injection visible.
This case terminated fatally on the eighth day after admission, and the
thirteenth after the date of the attack. The mode of dying was by apncea
An eruption, having the typhus characters, existed over the abdomen,
chest and extremities. Diarrhoea was not present, but slight meteorism and
tenderness on pressure. The respirations were accelerated, ranging from
40 to 56. Deep congestive redness of the surface existed, and the con-
junctiva was injected. There was present somnolency, muttering, and
toward the close of life, the power of articulation was lost. The pupils,
also, were somewhat dilated, and their mobility impaired. It is sufficiently
clear, from the foregoing summary of symptoms, that the case was one of
Typhus.
Note.—The clinical observations on fever which have been continued,
monthly, since August last, will, for the present, be suspended. The sub-
ject will be resumed and completed so soon as the writer’s leisure will al-
low him to prepare the remainder, but this, probably, will not be the case
for several months. The topics which remain to be considered are the
diagnosis, the identity of the two types, and the treatment.
				

